# Coumarins from Jinhua Finger Citron: Separation by Liquid–Liquid Chromatography and Potential Antitumor Activity

**DOI:** 10.3390/molecules28196917

**Published:** 2023-10-03

**Authors:** Chaoyue Wang, Jiangang Huang, Zhiling Zhou, Ping Xu, Jingyi Shi, Yushun Yang, Shengqiang Tong, Hongyu Hu

**Affiliations:** 1 Jinhua Advanced Research Institute, Jinhua 321015, Chinays_yang@nju.edu.cn (Y.Y.); 2 Xingzhi College, Zhejiang Normal University, Lanxi 321100, China; 3 College of Pharmaceutical Science, Zhejiang University of Technology, Hangzhou 310014, China

**Keywords:** Jinhua fingered citron, coumarins, liquid–liquid chromatography, anti-tumor activity

## Abstract

In this paper, liquid–liquid chromatography was introduced for the first time for the separation of fingered citron (*Citrus medica* L. var. *sarcodactylis* Swingle). The fingered citron cultivated in Jinhua is of significant industrial and medicinal value, with several major coumarin compounds detected in its extract. Therefore, further separation for higher purity was of necessity. A preparative liquid–liquid chromatographic method was developed by combining two elution modes (isocratic and step-gradient) with selection according to different polarities of the target sample. Five coumarin derivatives—5,7-dimethoxycoumarin (52.6 mg, 99.6%), phellopterin (4.9 mg, 97.1%), 5-prenyloxy-7-methoxycoumarin (6.7 mg, 98.7%), 6-hydroxy-7-methoxycoumarin (7.1 mg, 82.2%), and byakangelicol (10.5 mg, 90.1%)—with similar structures and properties were isolated on a large scale from 100 mg of petroleum ether (PE) extract and 100 mg of ethyl acetate (EA) extract in Jinhua fingered citron. The productivity was much improved. The anti-growth activity of the isolated coumarins was evaluated against three cancer cell lines (HeLa, A549, and MCF7) with an MTT assay. The coumarins demonstrated potential anti-tumor activity on the HeLa cell line, with 5,7-dimethoxycoumarin in particular exhibiting the best anti-growth activity (IC_50_ = 10.57 ± 0.24 μM) by inhibiting proliferation. It inhibited colony formation and reduced the size of the tumor sphere in a concentration-dependent manner. The main mechanism was confirmed as inducing apoptosis. This work was informative for further studies aimed at exploring new natural-product-based antitumor agents.

## 1. Introduction

**Figure 1:**
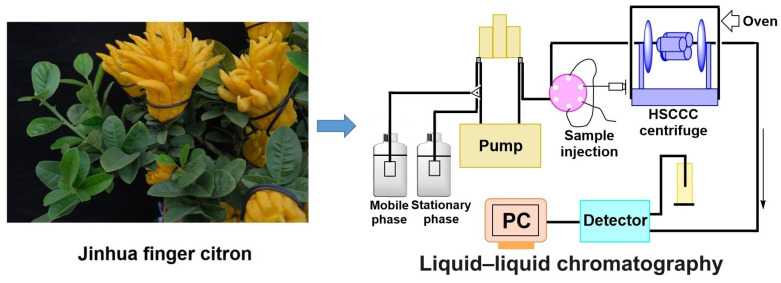
The Jinhua finger citron extract was separated by liquid–liquid chromatography to analyze the main coumarins.

Fingered citron, originated from India, is the fruit of *Citrus medica* L. var. *sarcodactylis* Swingle [1]. Since its introduction to China, it has been primarily cultivated in the middle and lower regions of the Yangtze River [2]. Cultivated in the four distinct seasons and in warm, humid, and sunny climates in the Jinhua area (Zhejiang, China), the fingered citron in Jinhua with a bright orange color and rich flavor (as shown in Figure 1) has been praised as “Celestial Fruit, Exotic Plant”. In traditional culture, the fingered citron was commonly adorned with pomegranates and peaches, symbolizing various blessings, including abundant offspring and longevity. It was revered as a symbol of good fortune and used in ancient times to ward off evil spirits and demons. The medicinal properties of fingered citron have been documented in notable works: the functions of regulating qi flow for eliminating phlegm, relieving cough, and reducing flatulence were recorded in the Ben Cao Gang Mu (Compendium of Materia Medica) [3]; the functions of nourishing the liver and warming the stomach, stopping vomiting, and eliminating upset stomach was recorded in Dian Nan Ben Cao (Herbal Medicines of Southern Yunnan) [4]; the functions of stopping vomiting and tonifying the spleen were recorded in Ben Cao Cong Xin (New Compilation of Materia Medica) [5]. Fingered citron is considered both a valuable food source and an essential component of traditional Chinese medicine, as it is rich in flavonoids, polysaccharides, and volatile oils [6,7]. Extensive pharmacological studies have shown that fingered citron possesses antioxidant [8], antibacterial [8,9], anti-inflammatory [10], anti-tumor [11,12], hypoglycemic [13], and hypolipidemic properties [13,14]. Furthermore, its ornamental and economic value has greatly contributed to its demand in foods, spice making, cosmetics, and in medicinal fields. However, current research on fingered citron mainly focuses on its volatile oil [1,2,15,16], with limited attention given to its other active ingredients and corresponding separation and activity research. Therefore, further exploration and rational utilization of fingered citron could enhance its value as both a food and medicine commodity.

It was reported that coumarin exists widely in fingered citron [6]. Due to its small molecular weight, simple synthesis process, and various bioactivities such as antibiosis, antitumor effects, anti-HIV activity, and antioxidation, coumarin currently arouses interest among scholars worldwide [17]. In a study by Cui et al. [18], the chromatographic separation of the PE and EA extracts from the 95% ethanol extract of Sichuan fingered citron resulted in the identification of 11 components. Building upon the above work, Yin and his team employed silica and SephadexLH-20 gel column chromatography to identify six more compounds, namely 7-hydroxyl-6-methylesculetin, 7-hydroxycoumarin, 7-hydroxy-5-methoxy coumarin, coumaric acid, nomilin, and stigmasterol [19]. Accordingly, the application of the functional compounds relies on identification and productivity.

Limited literature exists on the separation of fingered citron extract. Previous studies have utilized techniques such as silica gel column chromatography and macro-porous adsorption resin separation; however, these methods are not suitable for large-scale separation and have inherent drawbacks, including high costs, low preparation amounts, complex processes, and sample loss due to irreversible adsorption of the stationary phase [20]. Consequently, present separation technologies for fingered citron are insufficient, with unclear advantages and disadvantages of alternate methods, hindering further exploration of its functional components. The introduction of a practical separation method is essential for improving the determination and productivity of the typical compounds.

In order to fully exploit and utilize plant resources, it is essential to apply and popularize the extraction and separation technology of functional ingredients found in natural plants [21]. It is of great significance for the modern leisure food industry, health care industry, pharmaceutical industry, and other industries related to the national economy and people’s livelihood [21]. Liquid–liquid chromatography is a high-efficiency continuous liquid–liquid partition chromatography technology that uses immiscible biphasic solvent to move through a spiral tube at high speed, and uses planetary motion to facilitate the rapid and efficient separation and preparation of target ingredients [22,23]. Notably, the stationary liquid phase in liquid–liquid chromatography overcomes the issues related to adsorption and contamination, and effectively mitigates decomposition and deactivation of active components [24]. With the continuous progress of liquid–liquid chromatography technology, more and more traditional Chinese medicines and natural products can be clearly separated, and active ingredients can be discovered [25]. However, there are no reports on the application of liquid–liquid chromatography to separate fingered citron extract so far. Remarkably, this study established a liquid–liquid chromatographic separation method for fingered citron PE extract and EA extract (Figure 1). In the study, 5,7-dimethoxycoumarin (compound **I**), phellopterin (compound **II**), and 5-prenyloxy-7-methoxycoumarin (compound **III**) were separated from PE extract, and 5,6-hydroxy-7-methoxycoumarin (compound **V**) and byakangelicol (compound **IV**) were separated from EA extract. The obtained compounds all showed much higher productivity through our method. These compounds were assessed for their potential anticancer properties by using an MTT assay, and the results showed that 5,7-dimethoxycoumarin (compound **I**) exhibited the best inhibitory effects on cellular activity. In addition, the inhibiting mechanism of 5,7-dimethoxycoumarin (compound **I**) was further investigated by detecting its effects on cell proliferation and apoptosis. This study offers valuable insights into fingered citron’s pharmacological efficacy, physical basis, and clinical applications. Additionally, the isolation and preparation process outlined in this study presents a straightforward and effective method for obtaining coumarin compounds. Ultimately, the results of this study provide a new perspective on the potential application of natural coumarins in cancer therapy.

## 2. Results

### 2.1. Selection of Biphasic Solvent System

The efficacy of liquid–liquid chromatographic separation often relies on the selection of an appropriate solvent system. To evaluate the partition performance of coumarin compounds present in the PE extract of fingered citron, the fundamental solvent system of PE-EA-CH_3_OH-H_2_O was evaluated by utilizing HPLC. As per the observations recorded in Table 1, the K value of compound **I** across biphasic solvent systems one, two, and three were not sufficiently high to achieve the desired separation results, a trend mirrored by the K values of compounds **II** and **III**. Moreover, the similar K values of compounds **II** and **III** in solvent systems one, two, and three would result in peak overlapping, which is undesirable. However, solvent system four displayed improved K values for compounds **I**, **II**, and **III** at 1.285, 2.165, and 3.067, respectively, indicating its suitability for further separation. In addition, the α value of the three compounds was found to be greater than 1.4, thereby ensuring absolute separation. Hence, the solvent system comprised of PE-EA-CH_3_OH-H_2_O (4:6:5:5, *v*/*v*) was chosen for effectively separating the PE extract.

The selection of a suitable solvent system for the EA extract proved to be a challenging task due to the complexity and diversity of its components. The composition and volume ratio of solvent system required careful consideration to ensure that the distribution of the compounds was even. Our study involved testing several solvent systems on a high-speed counter-current apparatus, including *n*-butanol-EA-H_2_O, PE-CH_3_OH-H_2_O, EA-CH_3_OH-H_2_O, and PE-EA-CH_3_OH-H_2_O. Unfortunately, the first three solvent systems showed unfavorable partition performance, with most of the sample concentrated in the same phase. After several iterations, our observations revealed that solvent systems with PE-EA-CH_3_OH-H_2_O (3:7:3:7, 3:7:4:6, 3:7:5:5, *v*/*v*) had the ideal two-phase ratio and provided the best partition performance for components with significant polarities. The strength of the mobile phase elution increased with methanol content. Thus, we selected the two-phase solvent system with PE-EA-CH_3_OH-H_2_O (3:7:3:7, 3:7:4:6, 3:7:5:5, *v*/*v*), with a large polar range for the separation of the EA extract.

### 2.2. Liquid–Liquid Chromatographic Separation

In liquid–liquid chromatographic separation, the most common practice is to use the isocratic elution mode with a constant solvent system proportion. This mode is suitable for separating compounds with a similar polarity. However, achieving the ideal separation effect for samples with varying polarities can be challenging when using a constant solvent system ratio. The gradient elution mode is advantageous, as elution strength increases with the change in mobile phase composition while keeping the stationary phase constant. Consequently, a higher peak capacity is achieved, which makes gradient elution ideal for separating samples with large polarity spans. Nonetheless, it is challenging to perform gradients in liquid–liquid chromatographic separation, since the change in the mobile phase breaks the two-phase equilibrium, resulting in the loss of the stationary phase. The stationary phase should not change more than 20% to maintain accurate results. In our laboratory, we utilized both conventional isocratic elution and a step gradient to separate PE and EA extracts based on their polarity characteristics.

**Figure 2:**
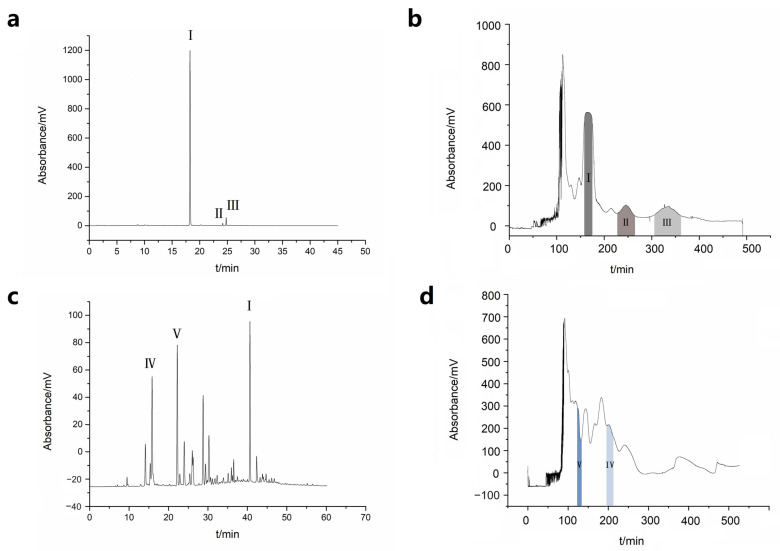
Chromatogram for HPLC analysis. HPLC conditions were presented in Section 3.4. (**a**) the PE extract of Jinhua fingered citron; (**c**) the EA extract of Jinhua fingered citron; Chromatogram for liquid–liquid separation of PE extract (**b**) and EA extract (**d**) of Jinhua fingered citron. Peak **I**: 5,7-dimethoxycoumarin; peak **II**: phellopterin; peak **III**: 5-prenyloxy-7-methoxycoumarin; peak **IV**: 6-hydroxy-7-methoxycoumarin; peak **V**: byakangelicol. Solvent system: (**b**): PE-EA-methanol-water (4:6:5:5, *v*/*v*); (**d**): PE-EA-methanol-water (3:7:3:7 for 0–100 min, 3:7:4:6 for 100–300 min, 3:7:5:5 for 300–500 min, *v*/*v*). Elution mode: (**b**): isocratic elution; d: step gradient elution. Sample injection: (**b**): 100 mg of PE extract dissolved in 10 mL of organic stationary phase; (**d**): 100 mg of EA extract dissolved in 10 mL of organic stationary phase. Revolution: 800 rpm. Flow rate: 1.5 mL/min. UV detection wavelength: 280 nm. Column temperature: 25 °C; Stationary phase retention: (**b**): 70.25%; (**d**): 40.38%.

The sample utilized for isocratic elution separation was the PE extract of Jinhua fingered citron. Figure 2a demonstrates that the extract consisted mainly of simple compounds. Liquid–liquid chromatographic separation of this extract was performed as seen in Figure 2b, resulting in one dominant peak and several smaller peaks. In contrast, the composition of the EA extract displayed a considerably more complex makeup with a broader range in polarity, as exhibited in Figure 2c. Consequently, gradient elution was deemed more appropriate for separating the EA extract by increasing the proportion of methanol to strengthen the elution strength of the mobile phase. The liquid–liquid chromatographic separation of the EA extract utilized the solvent system containing PE-EA-CH_3_OH-H_2_O (3:7:3:7 for 0–100 min, 3:7:4:6 for 100–300 min, 3:7:5:5 for 300–500 min, *v*/*v*), resulting in the separation chromatogram shown in Figure 2d, which plainly demonstrated the complexity of the EA extract with numerous peaks. Accordingly, the typical compounds were separated.

**Figure 3:**
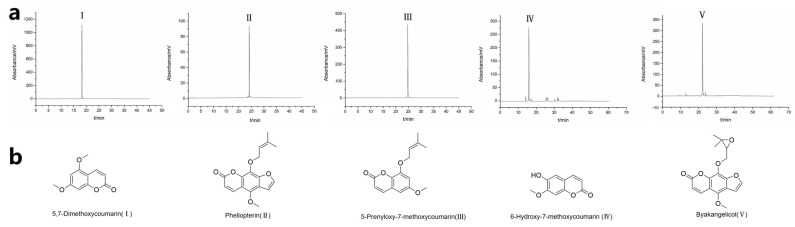
(**a**) Purified fractions collected from liquid–liquid chromatography. Peak **I**: 5,7-dimethoxycoumarin; peak **II**: phellopterin; peak **III**: 5-prenyloxy-7-methoxycoumarin; peak **IV**: 6-hydroxy-7-methoxycoumarin; peak **V**: byakangelicol. (**b**) The chemical structures of the isolated coumarins.

Upon the completion of HPLC fraction analysis, the fractions exhibiting high levels of purity were combined (Figure 3a). The resulting compounds obtained were as follows: compound **I** (52.6 mg, 99.6%), compound **II** (4.9 mg, 97.1%), compound **III** (6.7 mg, 98.7%), compound **IV** (7.1 mg, 82.2%), and compound **V** (10.5 mg, 90.1%). Subsequent identification of the structural composition was conducted using NMR and MS techniques, resulting in the determination of 5,7-dimethoxycoumarin (**I**), phellopterin (**II**), 5-prenyloxy-7-methoxycoumarin (**III**), 6-hydroxy-7-methoxycoumarin (**IV**), and byakangelicol (**V**). A depiction of the compound structures can be found in Figure 3b. The detailed characterization was as follows: Compound **I**: ^1^H NMR (600 MHz, DMSO-*d*_6_) *δ* 7.97 (d, *J* = 9.0 Hz, 1H), 6.57 (s, 1H), 6.49 (s, 1H), 6.17 (d, *J* = 9.6 Hz, 1H), 3.89 (s, 3H), 3.85 (s, 3H). ^13^C NMR (151 MHz, DMSO) *δ* 164.03, 160.78, 157.17, 156.67, 139.21, 111.08, 103.53, 95.45, 93.53, 56.76, 56.46. HRMS (Q-TOF *m*/*z*): Calculated for [C_11_H_11_O_4_]^+^: 207.0657, Found: 207.0632. Compound **II**: ^1^H NMR (600 MHz, Chloroform-*d*) *δ* 8.19 (d, *J* = 9.8 Hz, 1H), 8.09 (d, *J* = 2.3 Hz, 1H), 7.38 (d, *J* = 2.3 Hz, 1H), 6.34 (d, *J* = 9.7 Hz, 1H), 5.51 (t, *J* = 5.7 Hz, 1H), 4.75 (d, *J* = 7.2 Hz, 2H), 4.18 (s, 3H), 1.70 (s, 3H), 1.63 (s, 3H). ^13^C NMR (151 MHz, CDCl_3_) *δ* 160.59, 150.82, 145.10, 144.36, 139.71, 139.42, 126.94, 119.85, 114.94, 112.83, 107.61, 105.06, 70.41, 60.81, 29.71, 25.83, 18.09. HRMS (Q-TOF *m*/*z*): Calculated for [C_17_H_17_O_5_]^+^: 301.1076, Found: 301.1074. Compound **III**: ^1^H NMR (600 MHz, DMSO-*d*_6_) *δ* 7.99 (d, *J* = 9.7 Hz, 1H), 6.59 (d, *J* = 2.3 Hz, 1H), 6.54 (d, *J* = 2.2 Hz, 1H), 6.17 (d, *J* = 9.6 Hz, 1H), 5.49 (tt, *J* = 6.8, 1.4 Hz, 1H), 4.67 (d, *J* = 6.7 Hz, 2H), 3.85 (s, 3H), 1.77 (s, 3H), 1.74 (s, 3H). ^13^C NMR (151 MHz, CDCl_3_) *δ* 163.65, 161.76, 156.86, 156.28, 139.19, 118.68, 110.74, 104.29, 95.79, 92.70, 76.83, 65.65, 55.79, 25.79, 18.28. HRMS (Q-TOF *m*/*z*): Calculated for [C_15_H_17_O_4_]^+^: 261.1127, Found: 261.1123. Compound **IV**: ^1^H NMR (600 MHz, Chloroform-*d*) *δ* 7.64 (d, *J* = 9.4 Hz, 1H), 7.28 (s, 1H), 6.87 (s, 1H), 6.86 (s, 1H), 6.31 (d, *J* = 9.4 Hz, 1H), 3.97 (s, 3H). ^13^C NMR (151 MHz, CDCl_3_) *δ* 161.54, 152.83, 150.04, 146.35, 143.32, 113.56, 111.45, 107.95, 100.01, 56.36. HRMS (Q-TOF *m*/*z*): Calculated for [C_10_H_9_O_4_]^+^: 193.0181, Found: 193.0501. Compound **V**: ^1^H NMR (600 MHz, DMSO-*d*_6_) *δ* 8.19 (d, *J* = 9.8 Hz, 1H), 8.10 (d, *J* = 2.3 Hz, 1H), 7.39 (d, *J* = 2.3 Hz, 1H), 6.34 (d, *J* = 9.7 Hz, 1H), 4.46–4.39 (m, 2H), 4.17 (s, 3H), 3.63–3.66 (m, 1H), 1.13 (s, 3H), 1.04 (s, 3H). ^13^C NMR (151 MHz, DMSO) *δ* 160.15, 150.03, 146.77, 144.51, 143.62, 140.21, 127.30, 114.86, 113.00, 107.30, 106.10, 77.14, 76.28, 71.23, 61.28, 27.74, 24.86. HRMS (Q-TOF *m*/*z*): Calculated for [C_17_H_17_O_6_]^+^: 317.1025, Found: 317.1016 (Supplementary Material).

To the best of our knowledge, although representatives including compound **I** have been discovered by methods such as HPLC [26], the application of liquid–liquid chromatography has not been reported for the purification of fingered citron. Our methodology, which is founded on liquid–liquid chromatography, achieved exceptional results that included no irreversible adsorption, fast elution, higher recovery, and superior separation efficiency. Compared with the reported methods for the separation of compound **I** from natural products (the hexane extract of *Loricaria ferruginea*) using solid–liquid chromatography [27], our process yielded 103-fold higher production, required about one-third of the separation time, and consumed about one-fifth of the solvent, demonstrating its economic and environmentally-friendly nature. Likewise, liquid–liquid chromatography yields were about 5-fold higher for compound **II** [28], 300-fold higher for compound **III** [18], 320-fold higher for compound **IV** [29], and 500-fold higher for compound **V** [30]. It is worth noting that for the first time, compounds **I**–**V** were isolated from fingered citron using liquid–liquid chromatography. The much higher productivity may enhance the application value of typical compounds.

### 2.3. Biological Assays

The efficacy of isolated coumarin compounds extracted from Jinhua fingered citron was assessed on three different human cancer cell lines—HeLa (cervical cancer cell line), A549 (lung cancer cell line), and MCF-7 (breast cancer cell line)—utilizing an MTT assay with the positive control, cisplatin. The IC_50_ concentration required for 50% inhibition of cell viability was determined and presented in Table 2. The results indicated that the most promising antitumor activity was observed on HeLa cells specifically when compared with MCF-7 and A549. Additionally, MCF-10A cells (normal breast cell line) were utilized to evaluate the potential cytotoxicity of the isolated coumarin compounds. The results suggested that there was no antiproliferative activity observed on normal cell lines, thus indicating a favorable selectivity profile for these isolated coumarin compounds.

**Figure 4:**
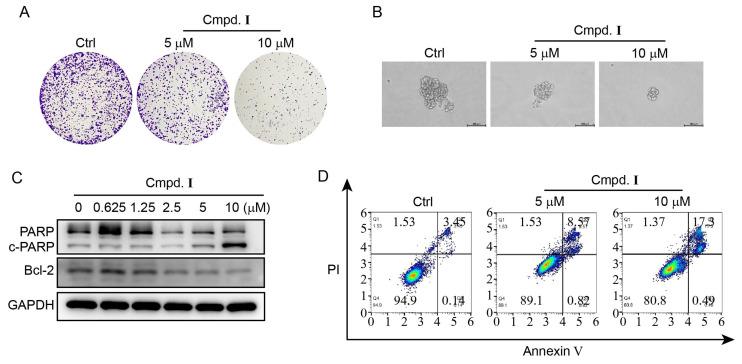
Compound **I** suppressed the growth of HeLa cells via inhibiting proliferation and inducing apoptosis. (**A**) The colony formation ability analysis of HeLa cells after treatment with different concentrations (0, 5, and 10 μM) of compound **I** for 7 days. (**B**) The tumor sphere formation ability analysis after treatment with different concentrations (0, 5, and 10 μM) of compound **I** for 10 days. (**C**) The western blot analysis on the protein level of PARP, c-PARP, and Bcl2 after treatment with various concentrations (0, 0.625, 1.25, 2.5, 5, and 10 μM) of compound **I**. (**D**) The flow cytometry analysis on the apoptosis percentage of HeLa cells after treatment with various concentrations (0, 5, and 10 μM) of compound **I**.

The findings presented in Table 2 indicate that compound **I** exerted a significant inhibitory effect on the growth of HeLa cells, as evidenced by the IC_50_ values of 10.57 ± 0.24 μM against HeLa cells. Based on the promising results, compound **I** was selected for further cellular experiments. We first tested the ability of compound **I** to inhibit the proliferation and stemness of HeLa cells using a colony formation assay and tumor sphere formation assay. The results showed that compound **I** inhibited colony formation and reduced the size of the tumor sphere in a concentration-dependent manner (Figure 4A,B). To assess the potential of compound **I** to induce apoptosis in HeLa cells, western blot analysis was conducted with several apoptosis markers, including PARP, cleaved PARP (c-PARP), and Bcl-2. The results indicated a dose-dependent increase in PARP cleavage and a decrease in the tested anti-apoptotic proteins (Figure 4C), thus highlighting the potential for compound **I** to inhibit tumor growth through its impact on apoptosis-related proteins. In addition, Annexin V-PI staining confirmed an increased rate of apoptosis in HeLa cells treated with compound **I**, in a concentration-dependent manner (Figure 4D). The previous reports indicated that compound I realized the inhibition on melanomas through the inhibition of mitogen-activated extracellular signal-regulated kinase (MEK) [31,32,33]. Therefore, the anti-cancer activity of compound **I** had potential in medicinal treatments, and the improvement on its productivity with liquid–liquid chromatography in this work was beneficial for realizing its practical utilization.

## 3. Materials and Methods

### 3.1. Apparatus

The TBE-200V type-J high-speed counter-current chromatographic apparatus (Tautochem, Shanghai, China) was utilized for this study. The separation column was comprised of a 1.6 mm internal diameter polytetrafluoroethylene tube with a 190 mL capacity. For this experiment, the optimal coil speed was 800 rpm. The sample loop had a volume of 20 mL. An MP-0106 constant flow pump was used to pump the stationary and mobile phase into the coil. Chromatograms were recorded using a 21C-B detector (Shanghai Kanghua Biochemical Instrument Manufacturing Factory, Shanghai, China) and SEPU 3010 workstation (Dell, Round Rock, TX, USA). The temperature of the column was regulated with the use of an SDC-6 thermostatic bath (Hinotek, Ningbo, China), whilst sample components were collected utilizing a BSZ-100 (Jiapeng, Shanghai, China) automatic component collector.

High-performance liquid chromatography (HPLC) was conducted using a Shimadzu LC-20AT HPLC Labsolution system (Shimadzu, Kyoto, Japan), consisting of a LC-20AD quaternary pump, a CBM-20A system controller, an SIL-20A autosampler, a CTO-20AC temperature controller, and an SPD-20A detector.

Mass spectrometry (MS) was conducted using an AB SCIEX Triple TOF 5600+ system (AB SCIEX Pte. Ltd., Los Angeles, CA, USA). ^1^H NMR and ^13^C NMR spectra were recorded on a Bruker Ascend TM 600 spectrometer (Bruker BioSpin, Rheinstetten, Germany).

### 3.2. Reagents and Materials

All of the organic reagents utilized in liquid–liquid chromatography was of analytical grade. The acetonitrile and methanol used in HPLC were of chromatographic grade. All chemical reagents were procured from Fangping Chemical Co., Ltd., (Hangzhou, China). The Jinhua fingered citron herbs, batch number 29 December 2020, were sourced from Zhejiang Jinshoubao Biotechnology Co., Ltd., (Jinhua, China) and authenticated by Chu Chu of Zhejiang University of Technology, confirmed as the dried fruit of *Citrus medica* L. var. *sarcodactylis* Swingle. The human cancer cell line HeLa, A549, and MCF-7 were all purchased from ATCC (Manassas, VA, USA).

### 3.3. Preparation of Crude Sample

To prepare the crude sample, 1.0 kg of fingered citron material (the fruit of the plant) was ground and subsequently immersed in 2.0 L of 70% ethanol solution for ultrasonic extraction for a period of 1 h. The resulting solution was concentrated under low pressure, yielding 196.0 g of brown viscous extract. This extract was then dissolved in 1.0 L of water, followed by repeated extraction with equal volumes of PE and EA. These layers were then concentrated to obtain 3.2 g and 6.6 g of PE and EA layer extracts, respectively.

### 3.4. HPLC Analytical Methods

HPLC was utilized to analyze the solvent extracts of Jinhua fingered citron and the components collected from liquid–liquid chromatography. The analysis was conducted using an H&E SP ODS-A C_18_ column (H&E Co., Ltd., Beijing, China, 250 mm × 4.6 mm, 5 μm). The PE extract and fractions obtained from the liquid–liquid chromatographic separation of the PE extract was analyzed under condition one, which involved a mobile phase composed of acetonitrile (A) and 0.10% ormic acid water (B). A gradient elution approach was employed as follows: 15–70% (A) in 0–20 min, 70–90% (A) in 20–21 min, 90–90% (A) in 21–30 min, 90–15% (A) in 30–35 min, and 15–15% (A) in 35–45 min. Similarly, the EA extract and fractions obtained from the liquid–liquid chromatographic separation were analyzed under condition two, which also involved a mobile phase composed of acetonitrile (A) and 0.10% ormic acid water (B). A gradient elution was used as follows: 15–25% (A) in 0–15 min, 25–30% (A) in 15–27 min, 30–50% (A) in 27–40 min, 50–90% (A) in 40–55 min, 90–15% (A) in 55–56 min, and 15–15% (A) in 56–63 min. The wavelength used was 326 nm. The flow rate was 1.0 mL/min. The column temperature was 30 °C. The injection volume was 20 μL.

### 3.5. Selection of Biphasic Solvent System

Various ratios of the PE-EA-methanol-water solvent system were selected based on the properties of the compounds and relevant reports on solvent systems utilized in separating coumarin compounds using liquid–liquid chromatography. Furthermore, an investigation on the separation time and partition coefficient was conducted.

The manner in which the partition coefficient K was obtained through HPLC involved the dissolution of roughly 1.0–2.0 mg of sample into a pre-equilibrium biphasic solvent system consisting of the upper (2.0 mL) and lower phase (2.0 mL). This was accompanied by an intense agitation of the glass-stoppered tube containing its contents. By subjecting an equidistant quantity of each phase to HPLC analysis, the area of the compound’s peak was captured for reference. Thereafter, the K value of the compounds was calculated using the following formula:

K = A_or_/A_aq_ (1)

Herein, A_or_ represented the peak area of compounds in the organic phase, and A_aq_ represented the peak area of compounds in the aqueous phase. The separation factor (*α*) was calculated using the following equation:

*α* = K_2_/K_1_(K_2_ > K_1_) (2)

### 3.6. Separation Procedure

The solvent system selected for this study was thoroughly mixed according to the prescribed ratio. Afterward, it was allowed to stratify to facilitate the separation into two distinct phases. Ultrasound degassing was employed to prepare the liquid–liquid chromatography solvent, and the tail-to-head elution mode was used for the separation. The stationary phase was first pumped at a rate of 20 mL/min, followed by the mobile phase at 1.5 mL/min while maintaining a rotation speed of 800 rpm. Equilibrium was established when the two phases were observed to flow continuously from the detector. About 100 mg of crude sample was injected into the column. The effluent was collected at 5 min intervals using an automatic partial collector, and the target components were obtained by drying under reduced pressure, as indicated by the chromatographic peak at a wavelength of 280 nm. The contents within the column were extracted by an air-compression pump, and the retention volume of the stationary phase was quantified.

### 3.7. Cell Culture and Treatment

The human cancer cell lines HeLa, A549, and MCF-7 were cultured routinely in a high-glucose Culbertson’s Modified Eagle Medium (DMEM) (Gibco Lab Inc., Grand Island, NY, USA) supplemented with 10% fetal bovine serum (Gibco Lab Inc., Grand Island, NY, USA) and 1% penicillin-streptomycin (Hyclone, Logan, UT, USA). The medium was changed daily, and cells were passaged at a density of 90%. Isolated coumarin compounds were dissolved in DMSO with a mother stock of 10 mM. Prior to cell administration, the compounds were freshly prepared in the specified concentration in medium.

### 3.8. Antitumor Activity

The MTT assay was utilized to measure the in vitro antiproliferation activity of the target compounds against HeLa, A549, and MCF7 human cancer cell lines. Standard procedures were followed, beginning with digestion of cells in good condition with 0.25% trypsin, and inoculation of 5000 cells per well in 96-well plates. After adhering to the surface, compounds were added in a fresh 200 μL medium per well, with the maximum concentration prepared initially and the double dilution method applied for the concentration gradient. Then, the medium containing these compounds was incubated in a cell incubator for 48 h. Accurate measurement of inhibition activity on cell proliferation was achieved by adding 20 μL MTT per well, followed by incubation at 37 °C for 4 h. The optical density (OD) of each hole at 490 nm was measured using a microplate reader (Thermo, MULTISKAN MK3, Thermo Fisher Scientific, Waltham, MA, USA) after the supernatant was removed and DMSO was added. The IC_50_ values of the target compounds were calculated using Graphpad Prism.

### 3.9. Colony Formation Assay

HeLa cells were digested and seeded in 12-well plates at a density of 1000 cells per well. Then, the plates were carefully transferred to the incubator, followed by incubation at 37 °C for 24 h. The adherent cells were treated with varying concentrations of the compound for 7 days, the medium was carefully removed, and the cells were stained with a 0.1% crystal violet solution for 10 min. The solution was removed and the plates were washed with PBS three times. The plates were dried at room temperature images of the cells were captured.

### 3.10. Tumor Sphere Formation Assay

Prior to digestion of the cells, the matrix gel was placed on ice to thaw, and the 24-well plates were placed in the incubator for preheating; the tubes and pipette tips were pre-cooled. Then cells were digested and adjusted to a density of 10^4^ cells/well with the medium, and the cell suspension was placed on ice for 2 min. The cell suspension was added to the matrix gel and mixed with gentle blowing, then 30 μL of the prepared matrix gel-suspension mixture was plated into each well of the pre-warmed 24-well plates, incubated at 37 °C for 30 min, and supplemented by 500 μL of the medium per well. They were continuously cultured for 10 days, and images of tumor sphere were obtained using inverted fluorescence microscope (Leica Microsystems, Wetzlar, Germany).

### 3.11. Flow Cytometry Assay

The cells were cultured in a six-well plate, with an approximate density of 1 × 10^5^ cells per well and subsequently treated with varying concentrations of the compound under investigation for 24 h. Following this, the cells were digested, collected, and subjected to staining for apoptosis detection, utilizing the Yeason kit (Yeasen Biotechnology Co., Ltd., Shanghai, China). The cell cycle status analysis was performed using the Beckman Epics Altra Culter (Brea, CA, USA), and data analysis was accomplished by means of Modfit 3.2 software (Topsham, ME, USA) after the cells were stained with DAPI for 15 min at room temperature.

### 3.12. Western Blot Analysis

The level of PARP, c-PARP, and Bcl-2 24 h after treatment with the hit compound was assessed using western blot analysis (Supplementary Material). The standard protocol involved treating cells with varying concentrations of the hit compound for 24 h, extracting whole cell lysates with the RIPA lysis buffer, and determining sample concentrations using the BCA kit from Sangon Biotech (Shanghai, China). Subsequently, 20.0 g of protein was loaded into a 10% gel and transmembrane PVDF (Millipore, Burlington, MA, USA, IPVH00010). After sealing with 5% skim milk for 1 h at room temperature, membranes were incubated with primary antibodies against PARP (CST, Danvers, MA, USA, 9542S), c-PARP, and Bcl-2 (Proteintech, Rosemont, IL, USA, 12789-1-AP) overnight at 4 °C. The membranes were then washed three times with TBST before incubating with anti-rabbit or anti-mouse antibodies conjugated with HRP tags for 1.5 h at room temperature. Finally, the blots were overlaid using the ECL detection kit and visualized under the ChemiDoc XRS+ system (Bio-Rad, Hercules, CA, USA). Anti-rabbit and anti-mouse IgG were purchased from Sigma (Burlington, MA, USA).

## 4. Conclusions

For the first time, a modern separation technique based on liquid–liquid chromatography was utilized to separate the components of Jinhua finger citron extract. Both conventional isocratic elution and a step gradient were employed based on the polarity characteristics of different substances extracted from Jinhua fingered citron. This led to the isolation of several high-purity simple coumarin compounds, including 5,7-dimethoxycoumarin, phellopterin, 5-prenyloxy-7-methoxycoumarin, 6-hydroxy-7-methoxycoumarin, and byakangelicol. The antitumor activity of these isolated coumarins was then evaluated, with some showing promising results on three different human cancer cell lines. Notably, 5,7-dimethoxycoumarin exhibited the highest inhibitory effect on the growth of HeLa cells. Colony formation and tumor sphere formation analysis found that 5,7-dimethoxycoumarin significantly inhibited HeLa cell proliferation and tumor stemness. Western blotting and Annexin V-PI staining provided evidence that 5,7-dimethoxycoumarin regulates the expression of apoptosis-related proteins and promotes apoptosis. Due to the significant presence of 5,7-dimethoxycoumarin as the primary active ingredient in Jinhua fingered citron, this compound is anticipated to be a potential anticancer candidate for further studies aimed at exploring new natural-product-based antitumor agents.

## Figures and Tables

**Table 1:** The distribution ratios and separation factors for coumarin compounds and impurities under the tested two-phase solvent system.

NO.	Solvent System		K_I_	K_II_	K_III_	α_II,III_
1	Petroleum ether-ethyl acetate-methanol-water	1:1:1:1	0.853	1.871	2.409	1.288
2		3:7:6:4	0.826	1.855	2.419	1.304
3		4:6:6:4	0.716	1.792	2.342	1.307
4		4:6:5:5	1.285	2.165	3.067	1.417

Equilibrium temperature: 25 °C. **I**: 5,7-dimethoxycoumarin; **II**: phellopterin; **III**: 5-prenyloxy-7-methoxycoumarin.

**Table 2:** The cytotoxicity of the candidate compounds **I**–**V** against HeLa, MCF-7, and A549 cancer cell lines.

Compounds	IC_50_ (μmol/L)		
	HeLa	MCF7	A549
I	10.57 ± 0.24	13.27 ± 0.38	14.13 ± 0.05
II	>100	>100	>100
III	12.30 ± 0.07	17.21 ± 0.26	19.87 ± 0.99
IV	22.18 ± 0.45	16.35 ± 0.63	21.79 ± 0.78
V	25.17 ± 0.22	26.48 ± 1.25	30.97 ± 0.71

**I**: 5,7-dimethoxycoumarin; **II**: phellopterin; **III**: 5-prenyloxy-7-methoxycoumarin; **IV**: 6-hydroxy-7-methoxycoumarin; **V**: byakangelicol.

## Data Availability

The data were available on request from the corresponding authors.

## Supplementary Materials

The following supporting information can be downloaded at: https://www.mdpi.com/article/10.3390/molecules28196917/s1, Figures S1–S10: Representative NMR spectra; Others: Original Western Blotting images.
