# Supplementary material for: Coumarins from Jinhua Finger Citron: Separation by Liquid–Liquid Chromatography and Potential Antitumor Activity
Source: Molecules. 2023 Oct 3;28(19):6917. doi: 10.3390/molecules28196917 (PMC10574065; doi:10.3390/molecules28196917)

## Supporting Information

# Coumarins from Jinhua Finger Citron: Separation by Liquid–Liquid Chromatography and Potential Antitumor Activity

**Chaoyue Wang [1,2,†], Jiangang Huang [2,†], Zhiling Zhou [1], Ping Xu [3], Jingyi Shi [1], Yushun Yang [1], Shengqiang Tong [3,*] and Hongyu Hu [2,*]**

1 Jinhua Advanced Research Institute, Jinhua 321015, China
2 Xingzhi College, Zhejiang Normal University, Lanxi 321100, China
3 College of Pharmaceutical Science, Zhejiang University of Technology, Hangzhou 310014, China
* Correspondence: sqtong@zjut.edu.cn (S.T.); huhongyu22@126.com (H.H.)
† These authors contributed equally to this work.

## Supplementary Figures

### Representative NMR spectra:

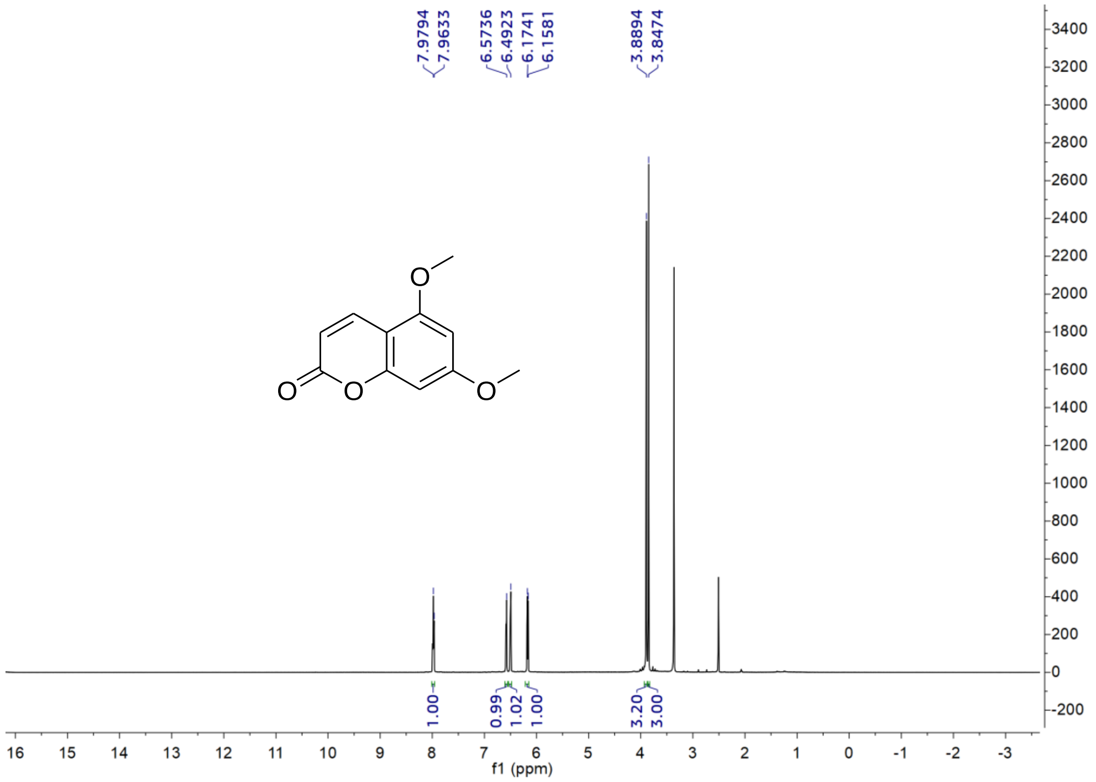


Figure S1. The $^1$H NMR spectrum of compound **I**.

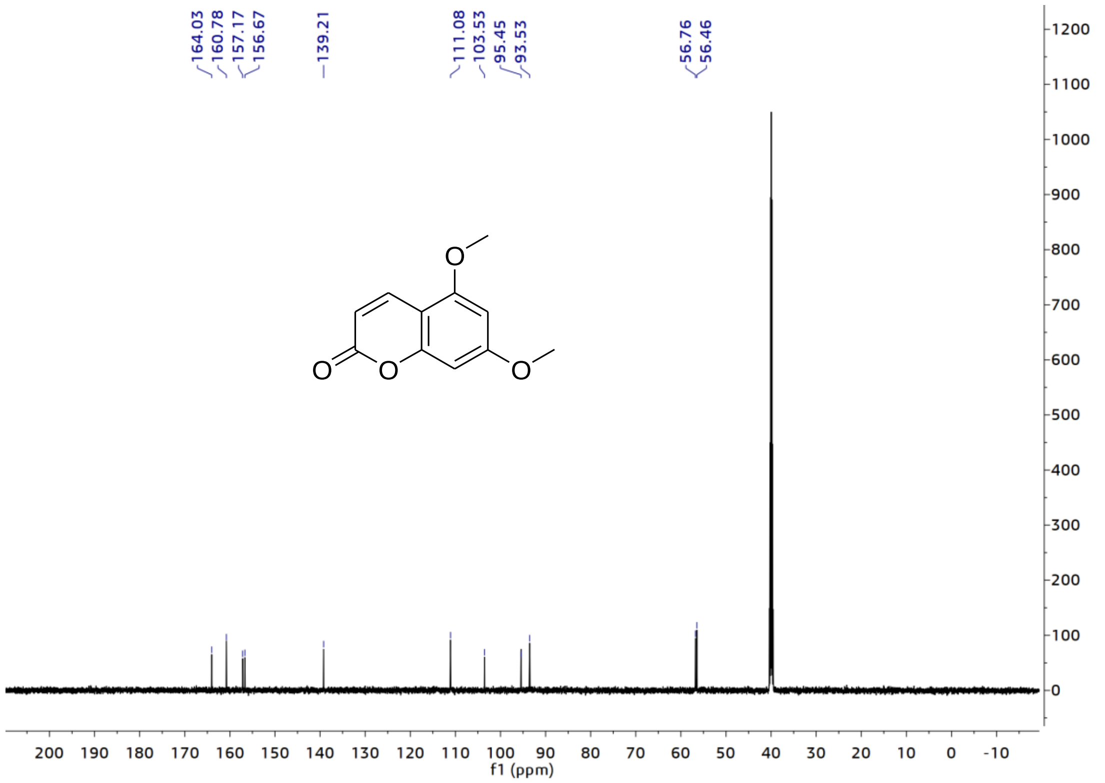


Figure S2. The $^{13}C$ NMR spectrum of compound **I**.

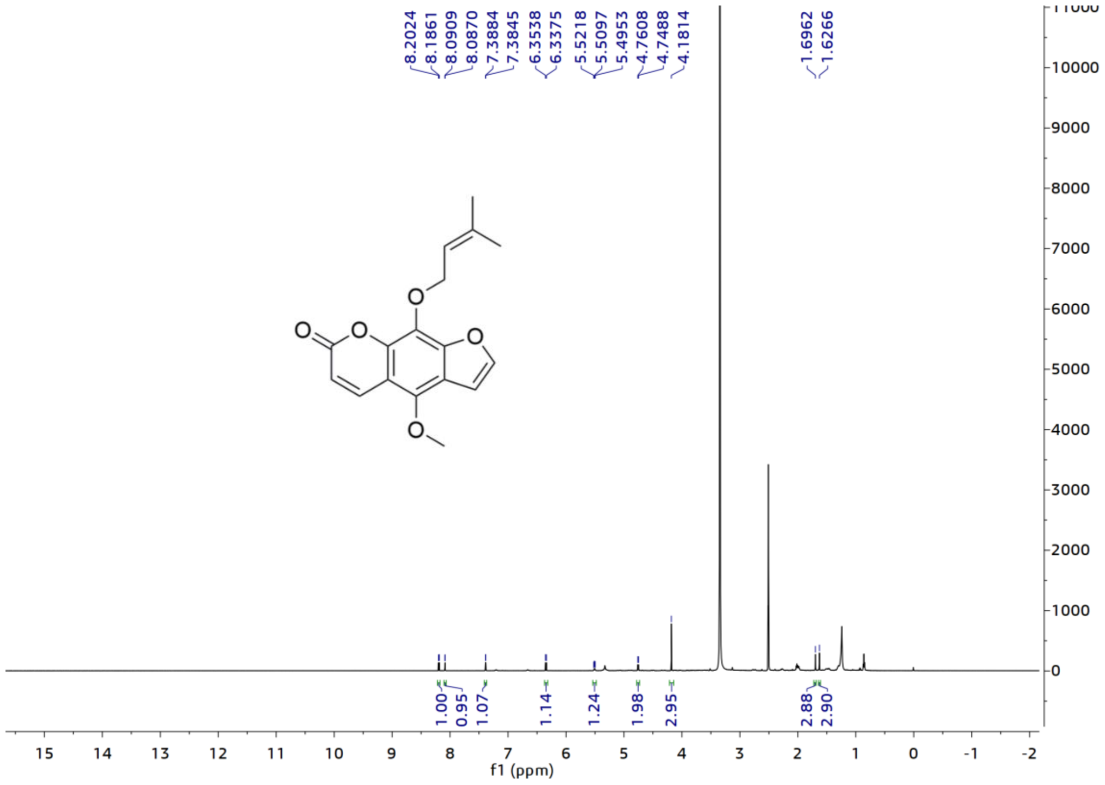


Figure S3. The $^{1}H$ NMR spectrum of compound **II**.

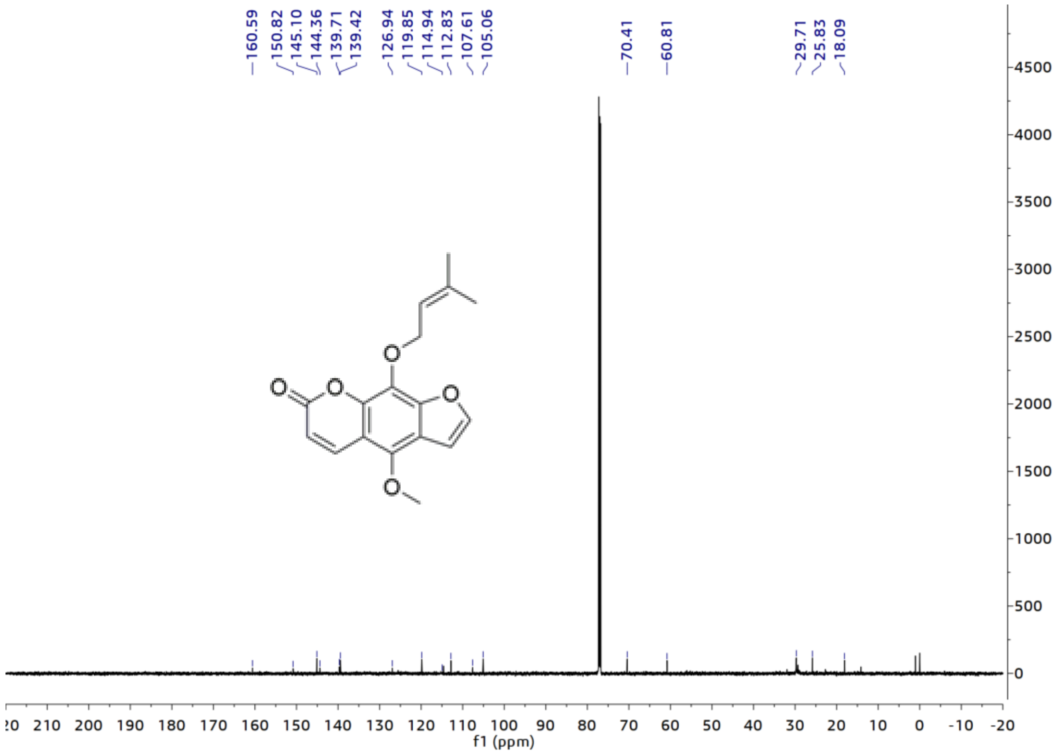


Figure S4. The $^{13}C$ NMR spectrum of compound **II**.

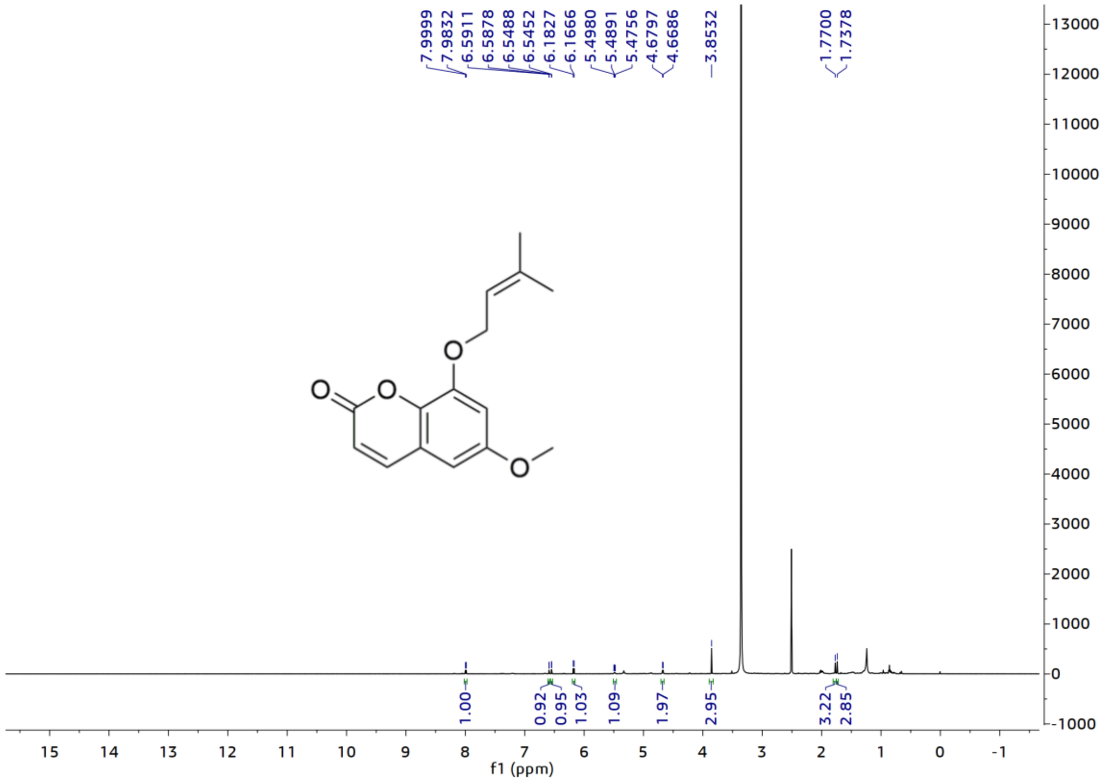


Figure S5. The $^{1}H$ NMR spectrum of compound **III**.

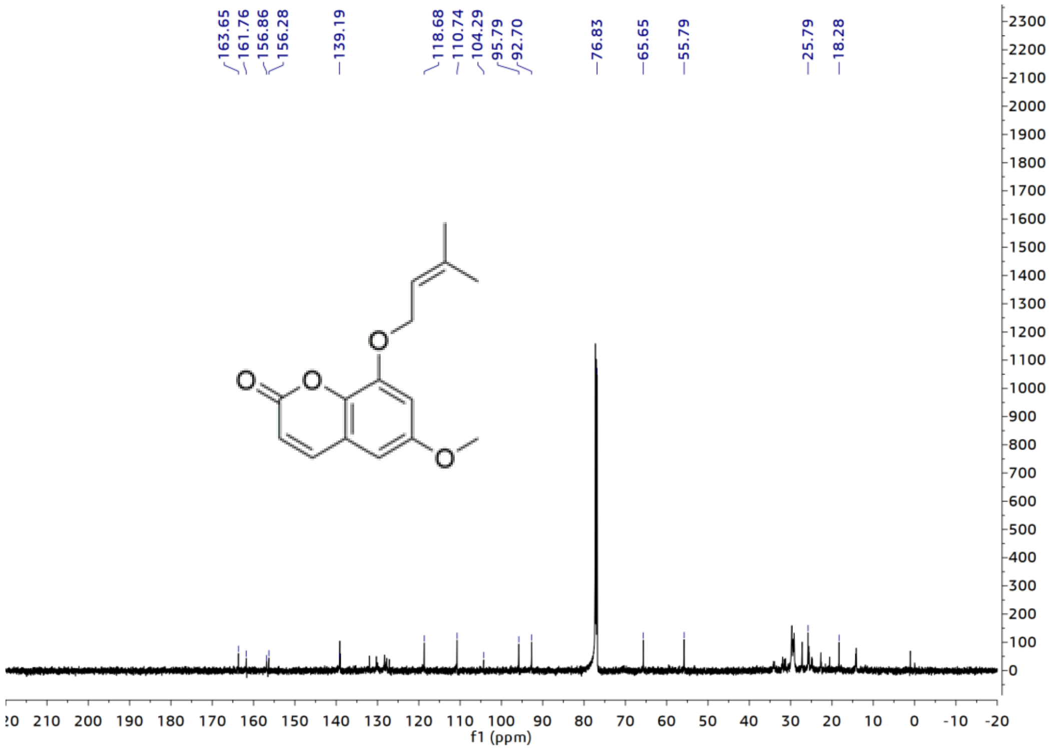


Figure S6. The $^{13}C$ NMR spectrum of compound **III**.

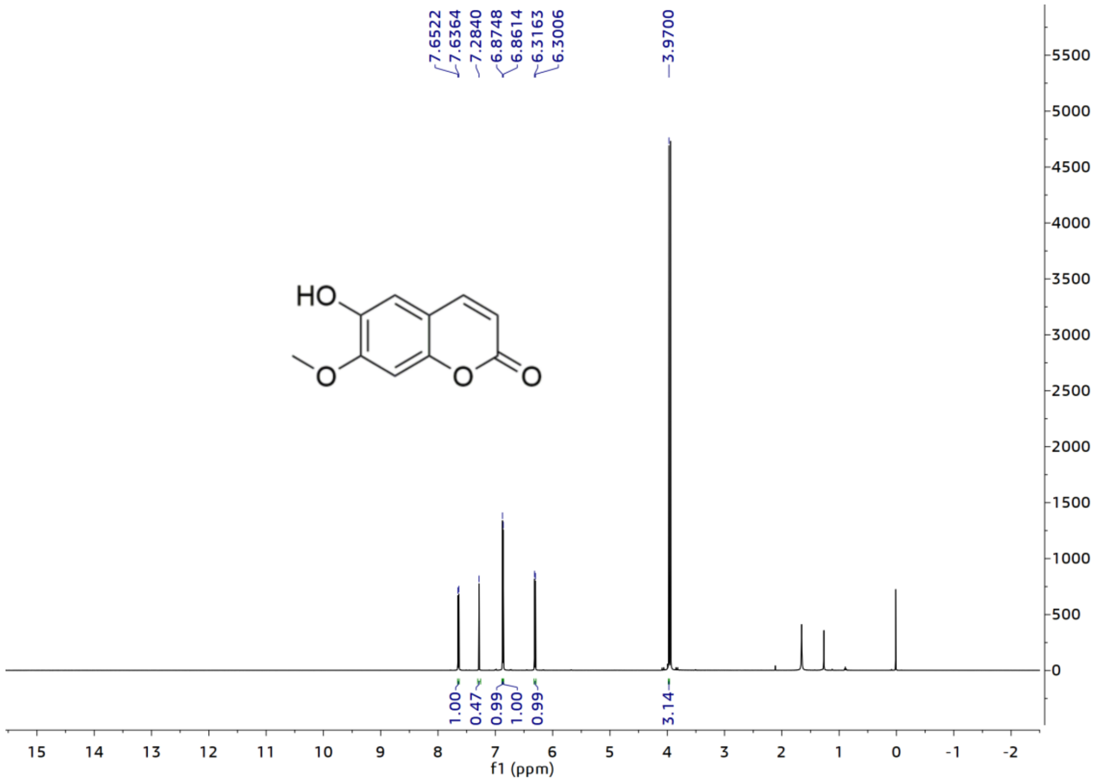


Figure S7. The $^{1}H$ NMR spectrum of compound **IV**.

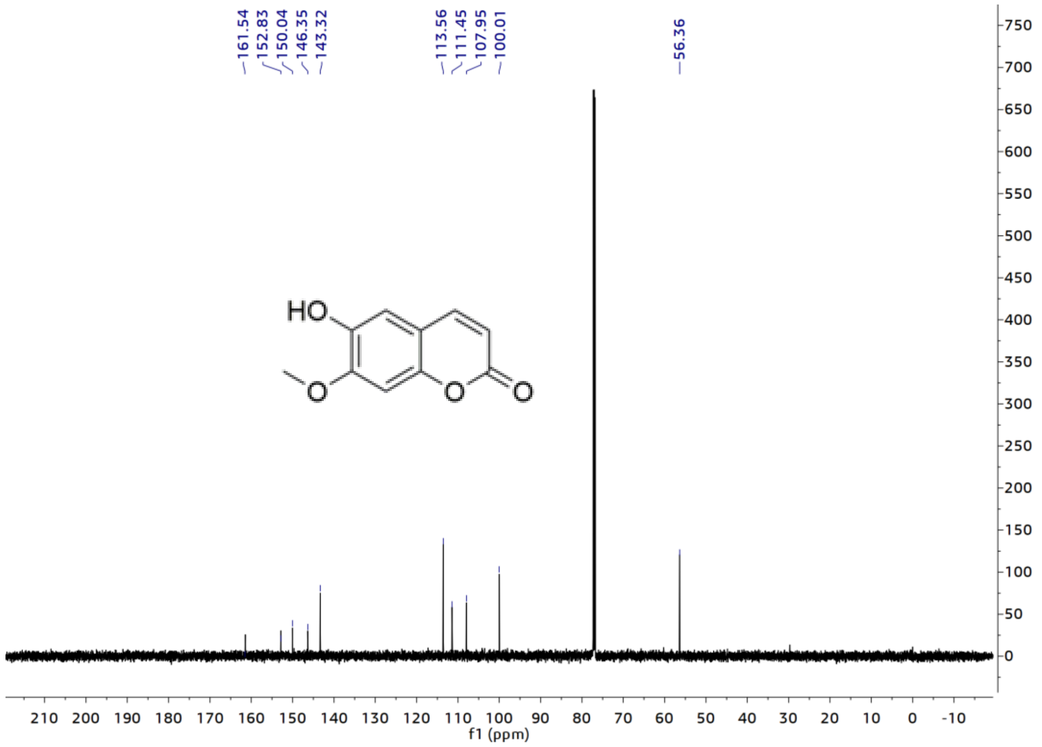


Figure S8. The $^{13}C$ NMR spectrum of compound **IV**.

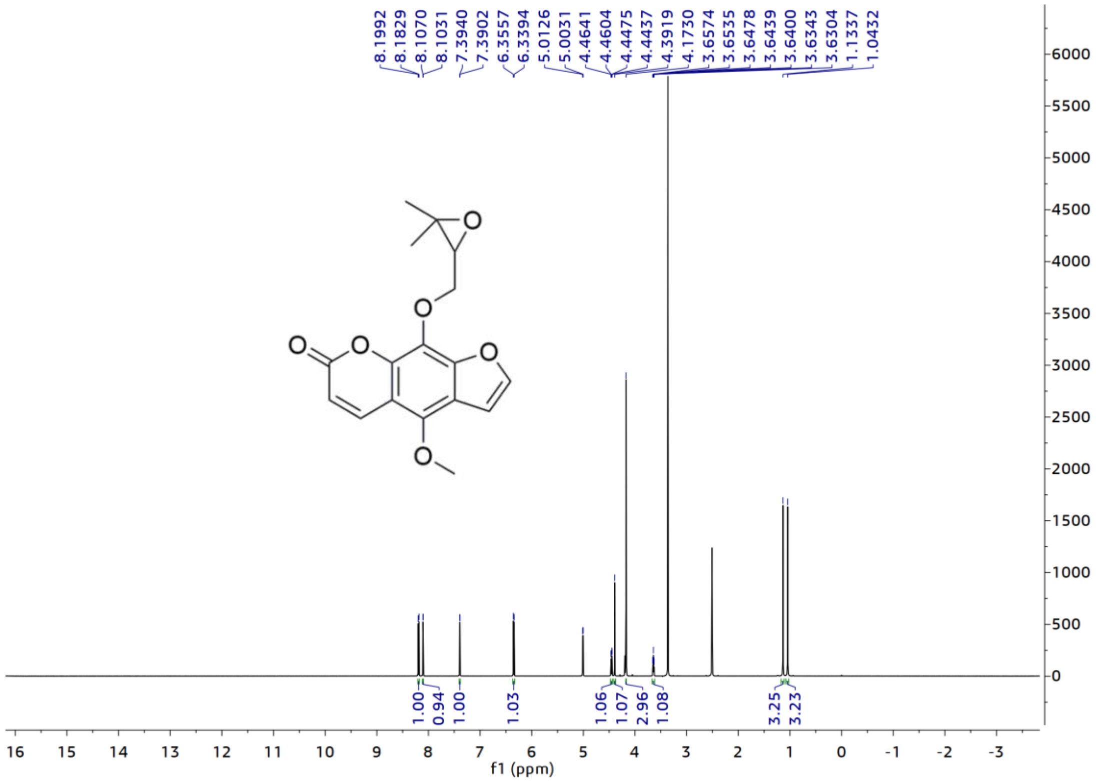


Figure S9. The $^{1}H$ NMR spectrum of compound **V**.

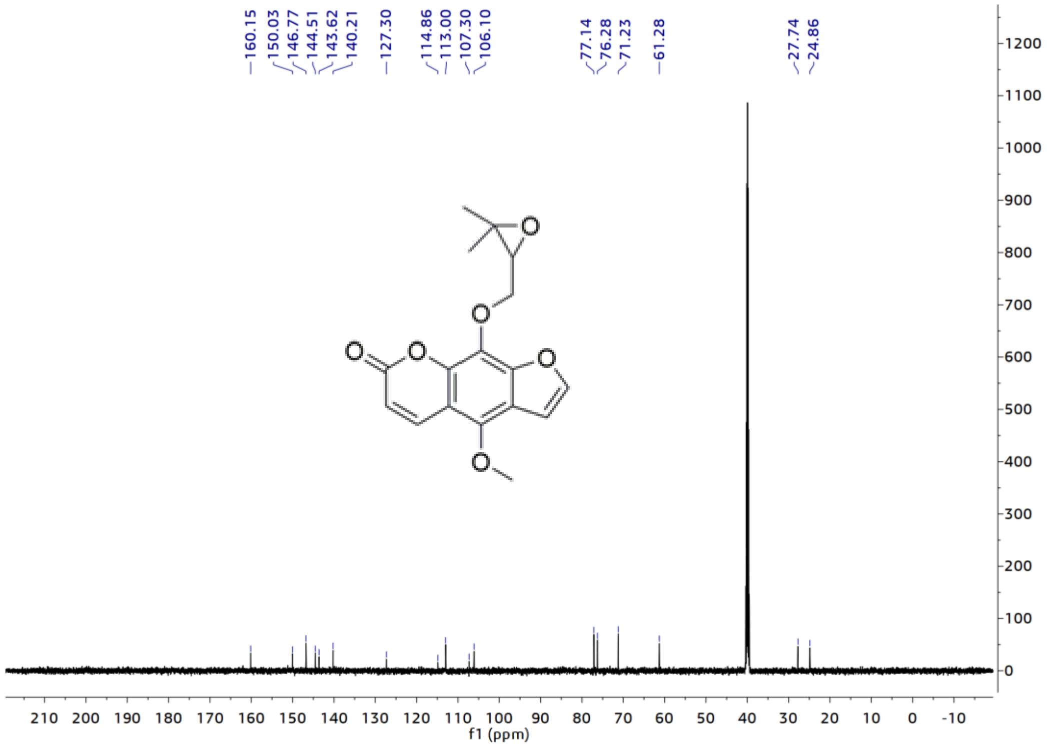


Figure S10. The $^{13}C$ NMR spectrum of compound **V**.

Original Western Blotting images:

PARP

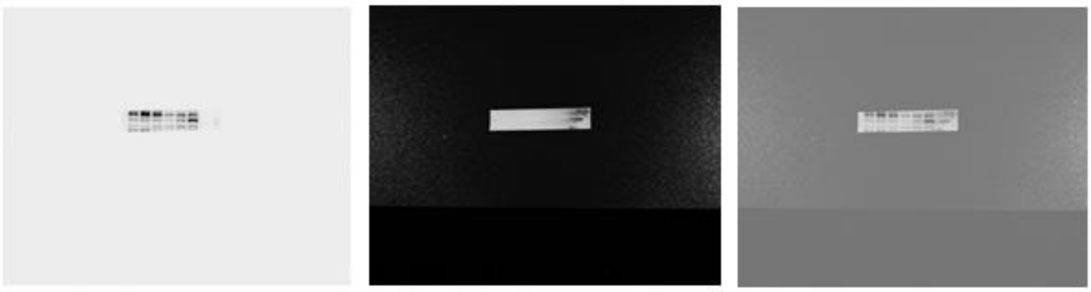

Bcl-2

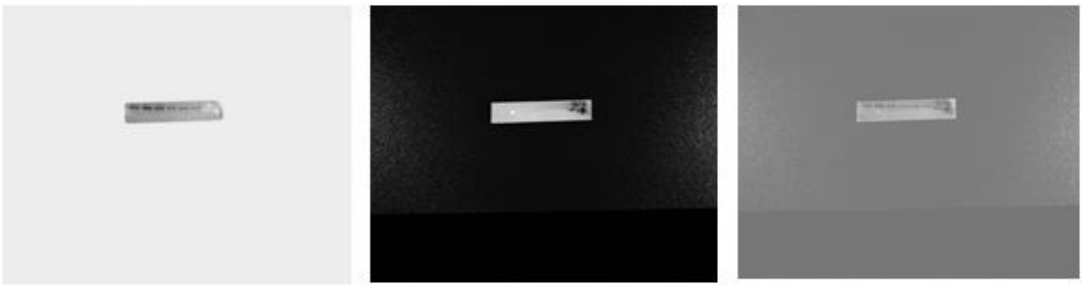

GAPDH

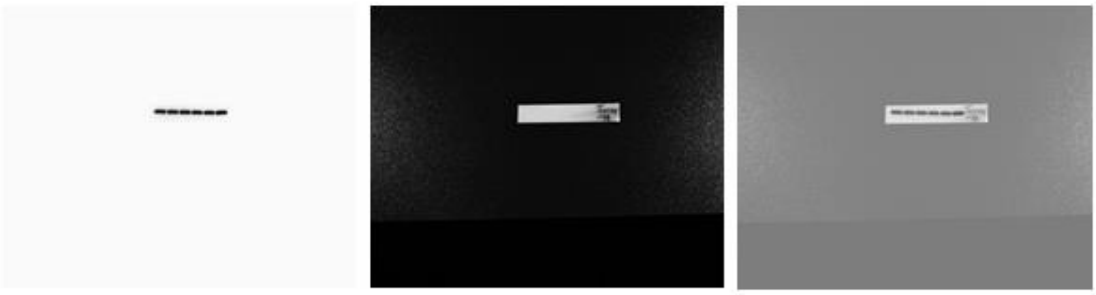

Supplement: Supplementary file 1 [file molecules-28-06917-s001.zip › molecules-2548170-supplementary.pdf]
